# Implementation of Image-Based Artificial Intelligence Is Associated with Increased Case Volume in a High-Acuity, 15-Room Cardiothoracic Operating Suite at a Tertiary Academic Hospital

**DOI:** 10.3390/jimaging12070283

**Published:** 2026-06-27

**Authors:** Ngoc-Anh A. Nguyen, Grace Lee, Sarah Sossong, Jannika V. Machnik, Sarah Pletcher, Roberta Schwartz

**Affiliations:** 1 Center for Connected Care, Innovation & Implementation—Research, Houston Methodist Hospital, Houston, TX 77030, USA; 2 Houston Methodist Academic Institute, Houston Methodist Hospital, Houston, TX 77030, USA; 3 Department of Medicine, Houston Methodist Hospital, Houston, TX 77030, USA; 4 Department of Surgery, Houston Methodist Hospital, Houston, TX 77030, USA

**Keywords:** image-based artificial intelligence, computer vision, operating room efficiency, surgical case volume, synthetic control, difference-in-differences, cardiothoracic surgery

## Abstract

Background: Operating rooms generate substantial visual data that is rarely captured systematically. Image-based AI (IBAI) systems using computer vision offer a new approach to real-time perioperative workflow monitoring, but evidence of their impact on surgical case volume remains limited. The aim of this study was to evaluate the association between deployment of an IBAI system and monthly surgical case volume in a high-acuity cardiothoracic operating suite, using synthetic control with difference-in-differences estimation. Methods: We deployed an IBAI system with wall-mounted cameras and a YOLO-based (You Only Look Once) object detection model coupled with a transformer-based event detector in a 15-room cardiothoracic suite at Houston Methodist Hospital (HMH), the tertiary academic hospital of Houston Methodist health system. The deployment was conducted under an IRB-determined quality improvement framework with patient consent for ambient video capture, defined retention limits, and restricted access to recordings. Over a 16-month period spanning 6 months pre-deployment and 10 months post-deployment, the system monitored 5417 surgical cases and automatically detected additional perioperative events including patient entry, draping, and room turnover. Using a synthetic control methodology, we compared post-deployment outcomes at the intervention site against a weighted combination drawn from a pool of 11 Houston Methodist sites that did not yet implement IBAI (116,098 cases across the comparison sites; 121,515 cases in the full analytic dataset). Results: The synthetic control analysis with difference-in-differences estimation showed a statistically significant increase of approximately 25 cases per month (95% CI 8.3 to 41.0; *p* < 0.01; Bonferroni-adjusted *p* < 0.05), corresponding to a 7% increase in monthly case volume relative to baseline. Conclusions: Our findings suggest that IBAI can meaningfully improve OR efficiency and support data-driven perioperative management. Future work should evaluate whether case volume gains generalize across other surgical specialties, assess changes in operational outcomes such as turnover time and first-case on-time starts, and examine clinicians’ perceptions of IBAI.

## 1. Introduction

A significant reduction in elective surgery case volume was observed worldwide at the peak of the COVID-19 pandemic, raising concerns about a global surgical backlog during the recovery period [1,2,3,4,5,6]. While some studies suggest that surgical case volume has rebounded to pre-pandemic levels [7], this trend is not uniform across specialties and further varies by care settings (ambulatory vs. inpatient), types of care facilities (private, public, or military), and geographic locations [8,9]. This phenomenon underscores the unprecedented urgency for healthcare systems to maximize operating room capacity post-pandemic to prevent further surgical delays and associated adverse clinical outcomes [10,11,12,13,14,15].

Previous efforts to improve operating room (OR) efficiency have focused on identifying specific perioperative events thought to cause bottlenecks and delays. Lean Six Sigma (LSS) methodology [16] has shown success by using process improvement tools, such as value stream mapping, spaghetti diagrams, Pareto analysis, and DMAIC framework (Define, Measure, Analyze, Improve, Control). Tagge et al. used LSS to redesign their OR workflow at an academic children’s hospital and analyzed records of 612 surgical cases following the intervention [16]. With process changes that focused on implementing parallel processes, increasing real-time awareness of performance problems, and reducing non-value-added activities, they saw a decrease in turnover time, defined as time between patient OR departure and arrival of the next patient. Similarly, Cima et al. at the Mayo Clinic, Rochester, observed improvements in on-time starts and reduction in the number of cases past 5 pm with LSS, leading to increased efficiency and financial performance [17]. Other initiatives have targeted scheduling optimization with a designated team [18], anesthesia-specific changes [19], and documentation optimization for better measurement of OR performance [20] with varying outcomes. The studies by Tagge et al. and Cima et al. illustrate this pattern: gains were achieved through dedicated multidisciplinary process improvement teams, intensive workflow re-engineering, and ongoing performance measurement, all of which require substantial institutional commitment and continuous leadership engagement to maintain [16,17]. Across these efforts, improvements have generally been modest in magnitude, and have required dedicated process-improvement personnel and ongoing manual data collection and review to achieve and sustain.

These methods are limited in several ways. First, most of them rely on retrospective data collected and/or analyzed by human observers, which do not account for unexpected real-time events that differ between operating rooms and surgical cases. Second, their heavy dependence on additional OR personnel time and associated costs makes these methods inaccessible to health systems with fewer resources. Most importantly, there is still no standard approach to classifying and segmenting the entire surgical process, leading to imprecision across studies [21] and confusion about the definitions of target perioperative events. Recent advances in artificial intelligence (AI), particularly computer vision, offer a potential response to each of these limitations by enabling automated, continuous, and standardized capture of perioperative events directly from the operating room environment [22].

Image-based AI (IBAI) systems apply computer vision algorithms to live camera feeds in operating rooms to identify and track patients, staff, and equipment in real time, automatically detecting perioperative events as they occur [22,23]. By generating event data continuously and without dependence on staff entry, IBAI directly addresses the retrospective, observer-dependent, and non-standardized nature of prior measurement approaches. IBAI systems also extend beyond passive measurement: real-time visualization of OR schedules, predictive forecasting of case durations, block-release of unused allocated OR time, and automated notifications to staff create opportunities for active workflow management during the surgical day rather than retrospective process review [24]. To date, however, the IBAI literature has focused primarily on the technical performance of event-detection algorithms [22,23] and on the legal, ethical, and implementation considerations of deploying ambient video monitoring in surgical environments [24]. Whether IBAI deployment translates into measurable operational improvements at the OR-suite level, particularly for surgical case volume, remains insufficiently characterized.

In this study, we report the implementation of an IBAI platform in a 15-room cardiothoracic operating suite at a tertiary academic hospital and conduct an observational evaluation of its operational impact over a 10-month post-deployment period. We extend the existing IBAI literature in three ways. First, we evaluate operational outcomes, specifically monthly surgical case volume, rather than algorithmic detection accuracy. Second, we apply a quasi-experimental design (synthetic control with difference-in-differences estimation) that constructs a data-driven counterfactual from contemporaneous comparison sites within the same health system, addressing the limitations of simple pre-post comparison. Third, we evaluate IBAI in a high-acuity, complex surgical environment, complementing prior work conducted in higher-volume ambulatory and orthopedic settings. To our knowledge, this is the first study to estimate the effect of IBAI deployment on surgical case volume using a synthetic control methodology.

## 2. Materials and Methods

### 2.1. Setting

**Figure 1:**
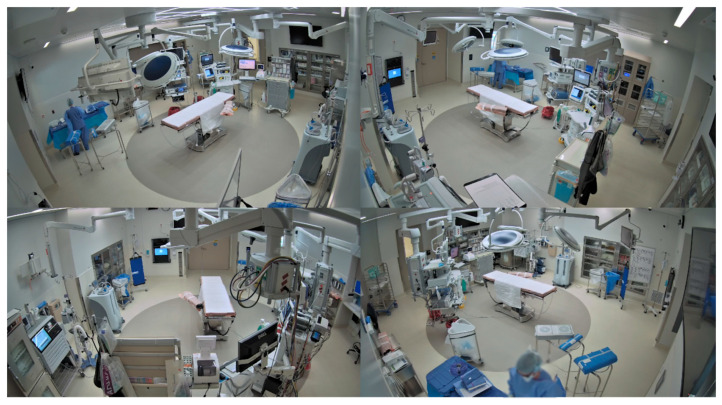
Camera installation and field of view. Wall-mounted cameras captured continuous image streams from multiple angles within each operating room. Image courtesy of Apella Technology.

Houston Methodist health system comprises one academic tertiary hospital, seven community hospitals, and one long-term acute care facility across the greater Houston region. An IBAI solution (Apella Technology, San Francisco, CA, USA) was implemented at a 15-OR suite at Houston Methodist Hospital, the flagship academic hospital, which performs approximately 4000 surgical cases per year. These ORs service cardiac surgery, thoracic surgery and thoracic transplant surgery. Each operating room was outfitted with four high-resolution wall-mounted cameras, with placement determined by room configuration to provide comprehensive image capture and minimize blind spots (Figure 1).

The cameras required a learning period, during which the system learned the objects within the room, analyzed data from each angle of the room, and used computer vision to identify the perioperative events occurring. Locations and actions were confirmed by a human-led quality control team.

The IBAI software was deployed at Walter Tower (WT) in December 2022. Camera installation began in September 2022 and system training began in October 2022, with full IBAI deployment in December 2022. Our analysis includes 6 months of pre-deployment data (June through November 2022) and 10 months of post-deployment data (December 2022 through September 2023).

### 2.2. Ethical Considerations and Data Governance

Implementation of an ambient video monitoring system in active operating rooms required an institutional governance framework addressing ethical oversight, patient consent, data retention, and access controls prior to deployment. The framework described below was developed at Houston Methodist Hospital in coordination with hospital legal services, the Operating Room Committee, and the Medical Executive Committee, and has been reported in detail previously [24].

The IBAI deployment was reviewed by the Houston Methodist Hospital Institutional Review Board and determined to be a quality improvement initiative classified as “Not Human Research,” for which a formal ethics approval submission was waived. The platform is compliant with the Health Insurance Portability and Accountability Act (HIPAA) and operates under hospital-specific policies developed to protect patient and staff confidentiality [24].

Patient consent for ambient video capture is incorporated into Houston Methodist’s general consent documentation, which was refreshed in 2022 to address digital health innovation technologies including IBAI. Signage informing patients and staff of IBAI utilization is posted in operating rooms where the platform is deployed [24].

Video footage is retained for a maximum of 30 days and audio for a maximum of 7 days, after which recordings are not stored and do not form part of the permanent medical record [24]. Access to intraoperative recordings, defined as the period from incision to skin closure, is restricted to three designated roles: the Chief Quality Officer, the Department Chair, and the Head of the Operating Room Committee. The surgeon is informed whenever an intraoperative recording is reviewed. Approval of additional users or new use cases is governed by the Operating Room and Medical Executive Committees [24]. Stakeholder engagement, governance evolution, and broader change-management considerations relevant to implementing AI-enabled technology in surgical environments have been described previously [24,25].

### 2.3. Features of IBAI

The key features of IBAI that were novel compared to the previous, traditional surgical workflow at Houston Methodist are as follows: 1. fragmentation of the surgical process into smaller, more identifiable events, 2. improved visualization of OR schedules using real-time tracking, and 3. automated, real-time documentation of perioperative events independent of staff-entered electronic health records (EHR).

**Figure 2:**
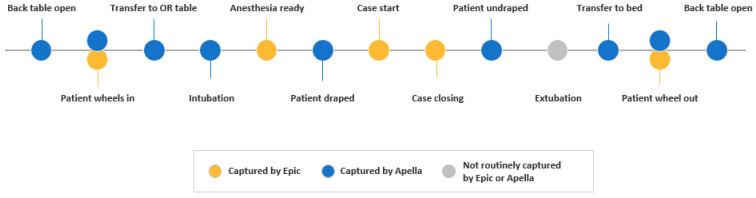
Additional perioperative events captured by IBAI System. The IBAI system can detect perioperative events that were either missing from the Epic EHR or not otherwise recorded during the case.

Our IBAI platform used computer vision algorithms to identify new key surgical events that were essential to analyzing OR methods but that had never been captured before (Figure 2). For example, the new “Patient Draped” event was recorded between the existing “Anesthesia Ready” and “Case Start” events. Previously, “Anesthesia Ready” and “Case Start” had been paired together, making cross-surgery comparisons difficult. By inserting “Patient Draped” between them, the workflow was divided into two distinct, measurable phases. With richer data now capturing the full perioperative process, the team leveraged IBAI to break surgeries down into shorter, more clearly defined phases.

**Figure 3:**
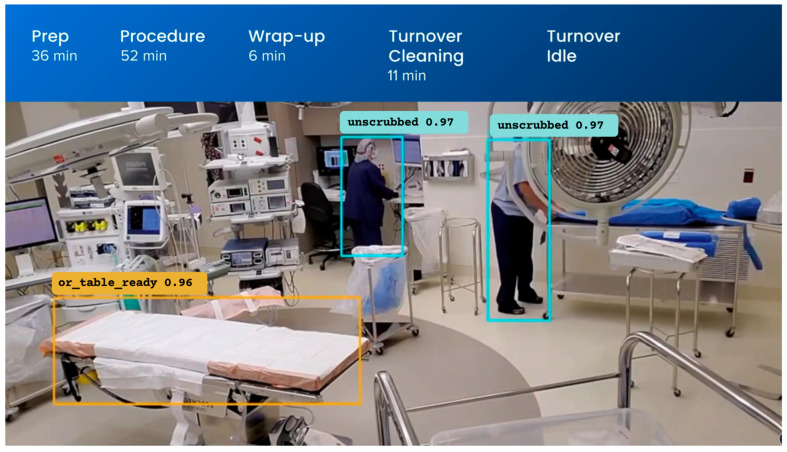
Computer vision object detection. The system identified and classified scrubbed and unscrubbed staff, patient draping status, and back table availability in real time. Image courtesy of Apella Technology.

The system used a YOLO-based (You Only Look Once) [26] model to identify patients, staff, and equipment from continuous camera streams (Figure 3).

The output informed a transformer-based event detector that analyzed temporal sequences of detected objects and their spatial relationships to predict key perioperative events in real time [23]. The system was trained on a global dataset comprising 137,517 surgical cases performed across 315 operating rooms by 1853 surgeons.

The object detection model was fine-tuned on annotated operating room images from multiple hospital sites and organizations to identify patients, scrubbed and unscrubbed staff, and surgical equipment, using a binary cross-entropy loss with standard optimization techniques and standard geometric and photometric data augmentation from the Ultralytics and PyTorch libraries. Further specifics of the model architecture and training, including the YOLO version, the backbone configuration, the structure of the transformer event detector, the train/validation/test split, and hyperparameter settings have not been disclosed by the platform vendor (Apella Technology). Because the present study evaluates the operational impact of the deployed system rather than its underlying machine learning methodology, these implementation details are not central to its contribution; readers are referred to Loo et al. [23] for a description of the platform.

Surgical domain experts annotated a random 10% sample of detected events, reviewed by a senior annotator to establish ground truth, with discrepancies resolved through consensus. In a separate validation study, the system achieved F1 scores, accuracy, precision, and recall values of 0.97 or greater across all events, with median time errors ranging from 6.4 to 16.9 s [23].

**Figure 4:**
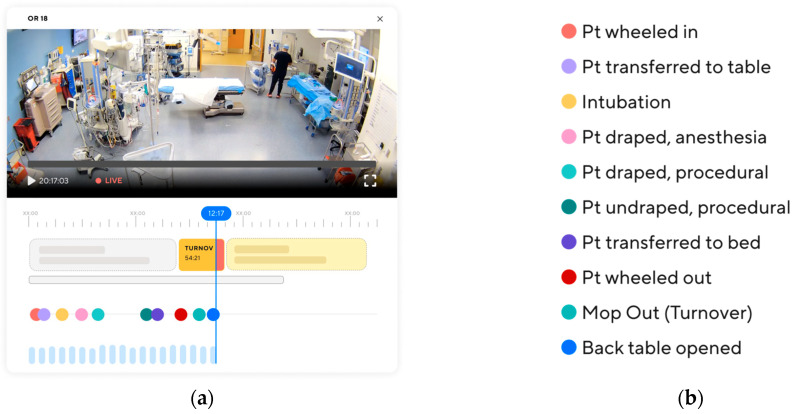
Real-Time OR event timeline (**a**) and legend (**b**). IBAI real-time OR schedule dashboard. Surgeon names and case details shown are synthetic and for illustrative purposes only. Image courtesy of Apella Technology.

After the learning period, management had access to real-time information and post-op reports on key surgical events within the OR, as well as a digital board displaying this information for easy viewing (Figure 4).

**Figure 5:**
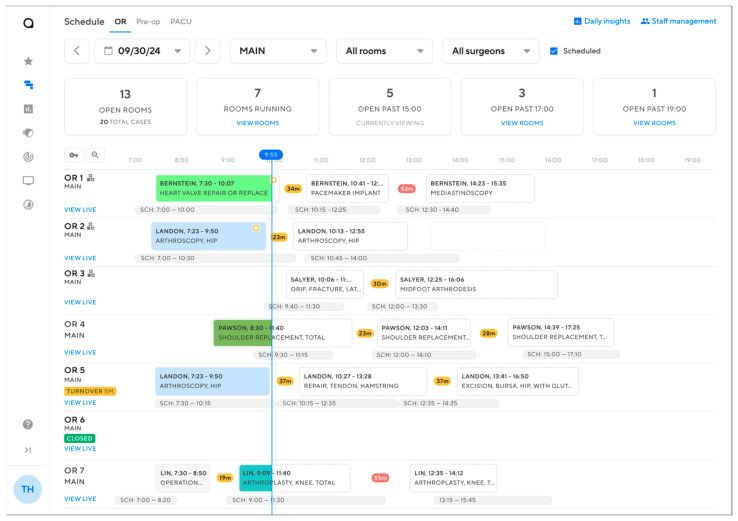
Sample real-time IBAI schedule dashboard. The dashboard displays live case progress and predicted case durations across all operating rooms, enabling dynamic decision-making around OR utilization. Surgeon names and case details shown are synthetic and for illustrative purposes only. Image courtesy of Apella Technology.

The IBAI schedule dashboard provided a real-time view of case progress and predicted case durations, enabling dynamic decision-making around OR utilization (Figure 5).

In addition to events already documented in the EHR, the IBAI system captured events not routinely recorded by clinical staff, due to the volume and pace of perioperative activity, including anesthesia and procedural drapes up and down, patient undraped, back table status, patient transfer to the OR table, and patient transfer to bed. Table 1 summarizes the full suite of platform tools available through the IBAI system, several of which directly supported the operational improvements observed in this study.

### 2.4. Synthetic Control Design

This is an observational study using a quasi-experimental design. The intervention evaluated in this study is the bundled deployment of the IBAI platform together with the operational and change-management activities that accompanied its rollout, including turnover-time review with OR management, housekeeping and infection control workflow reviews, anesthesia leadership reviews of induction and post-procedure timing, and surgeon notification protocols. Throughout this manuscript, references to “IBAI implementation” or “IBAI deployment” should be understood to refer to this bundle rather than to the technology platform in isolation. Estimating the effect of IBAI at WT requires constructing a credible counterfactual: what would have happened at WT in the absence of IBAI. This hypothetical scenario is central to causal inference but cannot be observed directly.

The gold-standard approach to causal inference, the randomized controlled trial, is not feasible in this study because IBAI was not randomly assigned across sites. A common non-experimental approach in the operational efficiency literature is to compare outcomes before and after the intervention. However, this method assumes that nothing else changed over time that could have affected outcomes, which is rarely a safe assumption in complex hospital environments.

Instead, we use synthetic control, a method common in economics literature. Rather than assuming that WT’s pre-IBAI trend would have continued unchanged, synthetic control builds a data-driven estimate of what would have happened at WT in the absence of IBAI by combining historical outcomes from other hospital sites within Houston Methodist that did not implement IBAI.

These comparison sites’ outcomes are weighted to match WT’s own outcome trends in the six months prior to IBAI adoption as closely as possible, using weights restricted to be non-negative and sum to one. This six-month window comprised all available pre-deployment operational data; because synthetic control estimation uses the pre-intervention period to estimate the donor weights, and a larger number of pre-intervention observations supports more reliable weight estimation [27], we matched on the full available pre-deployment window rather than a shorter one. This weighted combination of outcomes forms a synthetic control group, a tailored stand-in for WT in the absence of IBAI.

We construct a separate synthetic control group for each outcome of interest, since the best-matching combination of comparison sites may vary depending on the outcome. For example, the group used to estimate WT’s counterfactual case volume trend may differ from the group used to estimate its counterfactual overtime trend. We then compare the synthetic trajectory to WT’s actual trajectory over the 10 months following IBAI implementation to estimate impact.

This approach improves on both simple pre/post comparisons and benchmarks based on national or regional averages, which may differ from WT in important ways. By drawing only on other Houston Methodist sites that did not yet implement IBAI, but that share similar local labor markets, patient populations, and system-wide policies, synthetic control provides a more credible and contextually grounded estimate of what would have occurred at WT without IBAI.

### 2.5. Model Specifications

We estimated the effect of IBAI on case volume using two specifications: raw case counts and log-transformed case counts. The log specification reduces the influence of outlier months and allows interpretation of effects as approximate percentage changes. Because the optimal combination of donor sites may differ by specification, we constructed a separate synthetic control for each. The raw specification optimizes for matching absolute case counts, while the log specification optimizes for matching proportional trends. As a result, the algorithm may select different donor sites depending on whether absolute volume or relative growth patterns provide a closer pre-deployment match. We report results from both to demonstrate robustness. Synthetic control weights were estimated using the Python scpi_pkg package (version 2.2.7) to minimize the mean squared prediction error between WT and the weighted combination of donor sites during the pre-deployment period. To assess whether the effect estimated at Walter Tower was unusual relative to chance, we conducted an in-space permutation (placebo) test, re-estimating the synthetic control with each donor site treated in turn as the intervention site and comparing the ratio of post-deployment to pre-deployment root mean squared prediction error (RMSPE) at Walter Tower against the resulting placebo distribution.

For the raw case-volume specification, one of the 11 donor sites (HMH Dunn 6 OR) did not yield a converged synthetic control when assigned as the placebo-treated unit and was excluded from the placebo distribution; the permutation test for this outcome therefore comprised Walter Tower and the 10 donor sites that converged (11 units in total). This site, whose mean monthly volume differed substantially from the other donors, placed well below Walter Tower in a diagnostic run on volume-normalized series in which all sites converged, indicating that its exclusion does not affect the conclusion.

We estimated the impact of IBAI on each outcome using a difference-in-differences model in the form of a linear regression of each outcome on an indicator variable for WT, an indicator for WT interacted with an indicator for post-IBAI time period, and indicators for each month relative to launch of IBAI [28]. We estimated this model using the Python statsmodels package (0.14.6) [29]. The model was estimated on data with one row for WT and one row for the outcome-relevant synthetic control group for each month in the sample window, spanning the 6-month pre-deployment period (June through November 2022), and the 10-month post-deployment period (December 2022 through September 2023) for a total of 16 months. The reported coefficient is the interaction of the WT indicator with the post-IBAI time indicator, representing the causal impact of IBAI on that outcome for WT over the 10 months following go-live. To assess the parallel pre-trends assumption underlying this design, we regressed the pre-deployment gap between Walter Tower and its synthetic control on a linear time term over the six pre-deployment months, separately for each outcome; a slope not significantly different from zero indicates no systematic pre-deployment divergence.

The study was designed by the Houston Methodist investigative team. The original synthetic control and difference-in-differences analyses were conducted by Nate Hilger of Apella Technology, working only from the aggregated monthly site-level panel; he did not have access to patient-level data. These analyses were subsequently replicated independently by a Houston Methodist analyst using the same aggregated dataset. Case-level electronic health record data were extracted and held by Houston Methodist, and access to patient-level data was restricted to Houston Methodist personnel.

### 2.6. Data Collection

The data for this study were obtained from Houston Methodist’s electronic health record system and encompass all surgical cases performed across the Houston Methodist network between 1 June 2022, and 30 September 2023.

Cases were included if they met both of the following criteria: (1) non-emergent and scheduled in advance, and (2) performed at traditional operating room sites within the Houston Methodist network. Cases were excluded if they met any of the following criteria: (1) emergent or unscheduled, due to their inherent unpredictability and incompatibility with synthetic-control matching on scheduled volume; (2) performed at labor and delivery units, ambulatory surgery centers (ASC), or ambulatory surgery units (ASU), to maintain clinical comparability with the cardiothoracic case mix at Walter Tower. No additional cases were excluded for data-quality reasons; the data contained no records with missing timestamps, duplicate case identifiers, or zero or negative case durations.

The resulting dataset includes unique case identifiers, site and room information, scheduled and actual procedure start and end times, and other standard variables relevant to OR operations. These data enable the construction of our outcomes of interest. The primary outcome is monthly case volume, analyzed in both levels and logs. Secondary, exploratory outcomes are number of active ORs, cases per room, unplanned overtime per room (defined as the number of minutes the final case in an OR extends past 3:00 p.m. when it was originally scheduled to end by that time), and median late start minutes per case (including negative values for cases that begin earlier than scheduled). The 3:00 p.m. threshold reflects the end of scheduled prime operating hours at Walter Tower, after which unplanned operating room time carries disproportionate staffing and resource costs. Winsorization caps extreme outlier values at a specified percentile rather than excluding them, to reduce their undue influence on summary statistics. Two variables were winsorized at the 99th percentile: unplanned overtime past 3:00 p.m. (capped at 119 min) and actual case duration (capped at 523 min). The primary volume outcomes (case count and log case count) and median late start time were not winsorized. As a sensitivity analysis responsive to potential case-mix shifts, we additionally examined total operative minutes per month (the sum of wheels-in to wheels-out case durations) using the same synthetic control and difference-in-differences specification.

The final analytic dataset included 121,515 surgical cases performed across 12 Houston Methodist sites (individual operating room suites, several of which sit within the same hospital), comprising 5417 cases at WT and 116,098 cases across the 11 comparison sites. None of the 11 comparison sites adopted the IBAI platform at any point during the 16-month study window, eliminating the possibility of treatment contamination in the donor pool. Case-level data were aggregated to monthly site-level panels for use in the synthetic control and difference-in-differences analyses. Table 2 summarizes key characteristics of the analytic dataset.

## 3. Results

**Figure 6:**
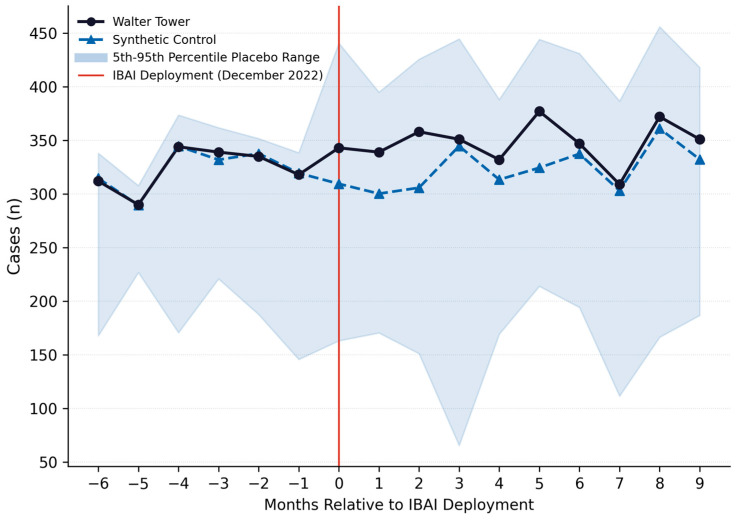
Monthly case volume at Walter Tower compared to the synthetic control group, from 6 months before to 10 months after IBAI deployment. The vertical line marks the December 2022 IBAI deployment date. The shaded band shows the 5th to 95th percentile range of the placebo distribution from an in-space permutation test, in which each donor site is treated in turn as the intervention site; inference is based on the ratio of post-deployment to pre-deployment prediction error. Walter Tower’s volume increased by approximately 7% relative to the synthetic control following deployment, consistent with the difference-in-differences estimate of approximately 25 additional cases per month.

We evaluated the effect of IBAI implementation on case volume and secondary operational outcomes using synthetic control methodology. Figure 6 shows how case volume evolved at WT compared to the synthetic control before and after WT launched IBAI. The synthetic control was constructed as a weighted average of five comparison sites, with the three largest weights assigned to HMH Main OR (0.36), HMH Dunn 6 OR (0.32), and HM San Jacinto OR (0.18) (Supplementary Table S1 outlines full donor weights by model specification). Figure 6 shows that WT’s case volume increased by approximately 7% relative to the synthetic control following IBAI deployment.

Consistent with this visual pattern, the difference-in-differences model estimates a statistically significant increase of approximately 25 cases per month, or roughly 7% against a baseline of approximately 339 cases per month (Table 3).

In the in-space permutation test, Walter Tower had the second-largest ratio of post-deployment to pre-deployment prediction error among the 11 units (Walter Tower and 10 donor sites; empirical *p* = 0.182). Because the comparison pool is small, the lowest attainable *p*-value is approximately 0.091, so the permutation result is directionally consistent with the difference-in-differences estimate but does not reach conventional statistical significance (Figure S2).

Among the secondary outcomes, median late start minutes per case decreased by 2.7 min (unadjusted *p* = 0.092); this result did not survive Bonferroni correction for multiple comparisons (adjusted *p* = 0.551) and should be regarded as exploratory. Unplanned overtime per room and open ORs per day trended in favorable directions but did not reach statistical significance over the 10-month post-deployment period. Monthly trajectories for all six outcomes (monthly case volume, log case volume, open operating rooms per day, cases per open operating room per day, unplanned overtime past 3:00 p.m., and median late start minutes per case) at Walter Tower and the synthetic control are presented in Figure S1.

To assess whether the increase in case volume reflected a shift toward shorter procedures rather than an operational gain, we conducted three additional analyses. Mean case duration at Walter Tower decreased modestly from 259.0 min (SD 144.6) pre-deployment to 245.7 min (SD 141.8) post-deployment, with median duration decreasing from 221.0 to 208.0 min (Supplementary Table S2). The procedural composition of the suite was stable across periods; the cardiothoracic, vascular, and thoracic service lines accounted for 86% of cases before deployment and 87% after, and the most frequent procedures were identical and in nearly the same rank order. In a sensitivity analysis using total operative minutes per month as a case-mix-insensitive outcome, the difference-in-differences estimate was not statistically significant (−1715 min per month; 95% CI −8122 to 4692; *p* = 0.575). These findings indicate that the volume increase occurred within a stable case mix and approximately constant total operative time, consistent with more completed cases per unit of operating room time.

The consistency of results across both raw and log-transformed specifications strengthens confidence in the robustness of the findings. Across all six outcomes, the pre-deployment gap between Walter Tower and the synthetic control showed no statistically significant linear trend, supporting the parallel pre-trends assumption. We also assessed pre-deployment fit directly using the root mean squared prediction error (RMSPE) between Walter Tower and its synthetic control over the six pre-deployment months, reported in the units of each outcome. The synthetic control fit Walter Tower closely for the primary case-volume outcomes and for open operating rooms per day (RMSPE of 3.341 cases for monthly case volume, 0.009 for log monthly case volume, and 0.122 rooms for open operating rooms per day), and less closely for cases per open operating room per day (0.721), unplanned overtime past 3:00 p.m. (2.607 min), and median late start minutes per case (2.475 min), consistent with the larger pre-period divergence noted for those secondary outcomes. The close pre-deployment fit for the primary outcomes supports the credibility of the synthetic control as a counterfactual for case volume [27].

## 4. Discussion

### 4.1. Principal Findings

To our knowledge, this study is the first to demonstrate an association between IBAI implementation and increased surgical case volume using the synthetic control methodology. The synthetic control analysis provided data that allowed us to estimate the proportion of growth attributable to IBAI, rather than relying on simple pre-post comparisons that cannot account for secular trends. As expected, the synthetic control group closely tracks WT before IBAI launched, suggesting it provides a plausible counterfactual for how WT’s volume would have evolved in the absence of IBAI.

IBAI supplied the granular operational data that enabled physician and administrative leadership to target inefficiencies and increase case volume. The intervention site used real-time accurate data provided to the appropriate parties with improvement-focused change management programs to create efficiency gains. Specifically, turnaround times and turnaround videos were reviewed with OR management. Housekeeping and infection control management also explored pathways for efficiency while improving quality. Anesthesia timing information was provided to anesthesia leadership who could work with outliers on induction and post-procedure in-room time. In a conference abstract presented at the 2024 European Society for Vascular Surgery Annual Meeting, Lengyel et al. reported that, in the Houston Methodist cardiovascular operating rooms, the system tracked patient entry and exit times with accuracy comparable to manual nursing documentation (median absolute error of 9.8 s versus 17.0 s for patient entry and 10.3 s versus 24.0 s for patient exit) and with significantly less variability in event registration [30].

The system offered several features that supported these improvements. IBAI fragmented the surgical process into discrete, measurable events, enabling more precise identification of inefficiencies. Real-time visualization of OR schedules allowed staff to identify available time from early case completions or cancelations. Automated text notifications informed surgeons when patients were draped or when their next case was approaching, reducing delays from surgeon unavailability.

Staff feedback from the implementation team supported the value of real-time visualization for situational awareness. Team members noted that the system’s live camera displays and case duration predictions made it easier to proactively identify and address issues and that it allowed for improved scheduling and available OR time. The data also made it more obvious which rooms were not being fully utilized and when, giving the team confidence to redeploy available room time and support more cases without overwhelming staff.

Implementing IBAI was a source of continued discussion in OR committees who wanted to ensure that the information was used for the purpose of efficiency rather than punitive action. Stakeholder engagement and transparent governance policies were essential to successful adoption. Our institution’s approach to addressing privacy concerns, consent, and data access has been described previously [24,25]. Initial skepticism among staff regarding ambient video collection diminished as the benefits of accurate, objective data became apparent. The information obtained through IBAI was viewed as trustworthy precisely because it was not subject to manual entry errors or recall bias.

The intervention was associated with a reduction in median late start minutes per case, suggesting that real-time visibility into case progress and surgeon notifications may also support more timely case initiation. This finding did not survive correction for multiple comparisons and should be interpreted as exploratory and hypothesis-generating, particularly given the 10-month follow-up period.

### 4.2. Limitations

Several limitations should be considered when interpreting these findings. First, attribution of the observed effect to IBAI remains challenging given that physicians joined and departed during the study period, and changes in room and block-time assignments occurred concurrently. All of these factors can affect volumes. Furthermore, as described in Section 2.4, the intervention evaluated in this study is the bundle of the IBAI platform together with the accompanying operational and change-management activities (turnover-time reviews, housekeeping and anesthesia workflow reviews, surgeon notification protocols). The current effect estimate therefore reflects the combined contribution of the technology platform and the change-management program, and cannot separately attribute the observed gains to one or the other component. We also do not have systematic data on co-interventions at the comparison sites during the study window; however, to the study team’s knowledge, no major concurrent workflow-improvement initiatives were undertaken at those sites during this period. We additionally note that for an unobserved co-intervention at a comparison site to have inflated the estimated effect, that site would have had to adopt changes that reduced its own case volume around the time of the IBAI launch; the more plausible scenario, in which comparison sites pursued their own efficiency gains, would bias the estimate toward the null and render the reported effect conservative. Nonetheless, the absence of a corresponding increase in staffing costs suggests that IBAI is likely an independent contributor to the observed gains.

Second, unplanned overtime did not reach statistical significance, which may reflect that case volume gains came primarily from better utilization of available OR time rather than from reducing end-of-day overruns. Alternatively, the 10-month follow-up period may have been insufficient to detect changes in overtime and other secondary outcomes. Although our analysis focused on case volume as the primary outcome, the system also captured data on late starts and turnover times, and formal evaluation of these secondary outcomes was beyond the scope of the present analysis. In addition, the unplanned overtime outcome is defined relative to a 3:00 p.m. threshold reflecting the end of scheduled prime operating hours at Walter Tower. Comparison sites may operate under different prime-time schedules, and the outcome definition does not account for this variation. Because the overtime result did not reach statistical significance and is not emphasized among our findings, we retained the site-specific 3:00 p.m. definition rather than constructing a site-adjusted measure of unplanned time past prime hours, which we note as a direction for future refinement.

These secondary outcomes can also be subject to month-to-month variability driven by case mix and staffing. Although monthly trajectories for all six outcomes are presented in Figure S1, longer follow-up will be needed to determine whether the patterns observed at 10 months represent durable changes. The post-deployment window was bounded by the available operational data, which were collected for implementation purposes rather than for this study; extending the follow-up period was therefore not feasible. Synthetic control and difference-in-differences methods are also best suited to bounded post-intervention windows, as treatment and comparison sites may diverge on unobserved factors over longer horizons. In addition, the 6-month pre-deployment window is relatively short for synthetic control estimation; we therefore report pre-period fit diagnostics and in-space permutation inference to characterize the credibility of the synthetic control given the available data, and we interpret the case-volume effect as associated with, rather than caused by, IBAI deployment.

Several methodological considerations also warrant discussion. The analytic pre-deployment window (June through November 2022) overlapped with the physical installation and learning phases of the IBAI system, as camera installation began in September 2022 and system training began in October 2022. Three of the six pre-deployment months therefore coincided with visible deployment activity in the operating rooms. Although intraoperative recordings were not yet active during this period, staff awareness of the impending deployment may have prompted anticipatory process changes or Hawthorne-style behavioral shifts that could partially attenuate the magnitude of the estimated post-deployment treatment effect.

To examine the sensitivity of our findings to this concern, we conducted a supplementary analysis restricting the pre-deployment window to June through August 2022, the three months preceding any visible installation or training activity (Supplementary Table S3). The estimated effect on monthly case volume remained positive in both the raw-count and log specifications. In the primary raw-count specification it was smaller than in the full window and no longer statistically significant, while in the log specification it remained statistically significant, although the near-perfect pre-period fit obtained in the three-month window is consistent with overfitting given the small number of pre-intervention observations. We therefore interpret the direction of the effect as robust to excluding the installation and learning months, while its magnitude and statistical significance are sensitive to the length of the pre-period and to the model specification. The six-month pre-deployment window thus represents a trade-off between excluding the installation period and retaining sufficient pre-intervention data for reliable synthetic control estimation [27]. An extended historical pre-period (for example, using 2021 data) could address this trade-off more directly but was not feasible, as operational data were available only from June 2022 onward.

We also observed a modest reduction in mean case duration at Walter Tower across the post-deployment period (approximately 5%). Because the service-line and procedure mix was stable across periods, this reduction is unlikely to reflect a shift toward different, shorter procedure types; we cannot, however, fully exclude finer-grained variation in case length within service lines.

Finally, the system’s full event-detection performance metrics (the F1, accuracy, precision, and recall values noted in Section 2.3) were established in a separate evaluation reported by Loo et al. [23], rather than through local validation on the Walter Tower cardiothoracic dataset. Because that evaluation was not specific to the Walter Tower cardiothoracic suite, differences in case mix, room configuration, and procedural complexity could affect detection accuracy in ways that have not been formally characterized. A separate prospective comparison of the platform against manual documentation in the Houston Methodist cardiovascular operating rooms reported high accuracy for patient entry and exit times [30]; that evaluation, however, was limited to patient entry and exit events and did not assess the full set of perioperative events detected by the system. Local validation across the complete event suite on the Walter Tower cardiothoracic dataset was therefore beyond the scope of this operational outcomes evaluation but represents an important direction for future work, particularly as IBAI deployment expands to other surgical specialties with distinct workflow patterns.

### 4.3. Future Directions

This study characterizes the operational outcomes associated with IBAI in a single high-acuity cardiothoracic suite, and several directions warrant further investigation. First, system-wide implementation across additional Houston Methodist hospitals and evaluation in other surgical specialties would establish whether the case volume gains observed here generalize beyond cardiothoracic surgery, where case complexity and duration differ substantially from higher-volume ambulatory settings. Second, the current analysis focused on case volume as the primary outcome; future work should formally evaluate turnover time, first-case on-time starts, and block utilization as primary endpoints, outcomes for which IBAI’s real-time event detection is particularly well suited. Third, a recent conference abstract by Lengyel et al. [30] suggests that AI-enhanced computer vision may offer more consistent and accurate tracking of OR events compared to manual documentation; peer-reviewed replication and extension of that work to additional sites and event types would strengthen the evidence base for broader adoption. Fourth, modules currently in development for block time release and room optimization represent a natural next step, with prospective evaluation needed to quantify their incremental contribution to efficiency gains. Finally, physician and staff perceptions of ambient video monitoring remain understudied; qualitative and mixed-methods research examining trust, governance preferences, and behavioral responses to real-time performance feedback will be essential to inform responsible implementation at scale.

## 5. Conclusions

Implementation of an IBAI system in a high-acuity cardiothoracic surgical suite was associated with a 7% increase in monthly case volume, based on synthetic control analysis. To our knowledge, this is the first study to evaluate the operational impact of IBAI using synthetic control methodology, providing a more rigorous estimate of effect than simple pre-post comparisons. These findings suggest that computer vision systems that capture granular perioperative events and present them through real-time visual dashboards can meaningfully improve OR efficiency. As health systems seek to address surgical backlogs and optimize limited operating room capacity, IBAI represents a promising tool for data-driven workflow management, with potential benefits extending to case scheduling and on-time start performance.

## Figures and Tables

**Table 1:** Apella image-based AI (IBAI) tools, functions, and deployment status at Walter Tower (December 2022 through September 2023).

Tool	Function	Used at Walter Tower (December 2022–September 2023)
Insights	An analytics tool that allows visualization of key operational data, such as first case on-time starts, turnover time, and case metrics.	Yes
Terminal cleans	Visibility into end-of-day OR cleaning status.	Yes
Highlights	Ability to flag and comment on specific video segments for review.	Yes
Live view	A livestream gallery providing visualization into every OR, with an overlay of case and room statuses generated by computer vision technology.	Yes
Schedule	Enhances the existing OR schedule with predictive forecasting and real-time event detection.	Yes
Pre/post-operative schedule views	Provides a chronological list of upcoming procedure starts and ends based on forecasted schedule.	Yes
Daily insights	Key operational metrics for the day to inform the morning huddle.	Yes
Boards	View of the day’s schedule and staffing designed for large displays to keep all staff informed.	No
Schedule assistant	Enables precise scheduling by recommending surgeon- and procedure-specific case durations.	No
Staff management	Calculates hourly staffing recommendations based on staffing ratios and predicted schedule.	Yes
Text notifications	Automated alerts for Patient Wheels In, Patient Wheels Out, and Patient Draped to keep surgeons and staff informed in real time.	Yes

IBAI: image-based artificial intelligence; OR: operating room [24]. Deployment status reflects active use at Walter Tower during the study window (December 2022 through September 2023), as reported by the implementation team. Tools listed as “No” were available on the platform but not in active use at this site during the study period. Adapted from [24].

**Table 2:** Summary Statistics, 1 June 2022–30 September 2023.

	Walter Tower	All Other Sites
Total cases	5417	116,098
Distinct sites	1	11
Mean case duration (minutes)	250.45	122.90
Avg distinct rooms per site	15.00	16.09
Mean monthly case volume (site-month avg.)	338.56	659.65
Mean log monthly case volume (site-month avg.)	5.82	6.33
Cases per room per day	1.31	2.24
Number of open rooms per day	8.52	10.13
Median late start minutes per case	0.00	7.00
Overtime minutes past 3 pm per room per day	9.52	7.48

Comparison sites exclude labor and delivery, ambulatory surgery centers, and ambulatory surgery units to ensure clinical comparability with the Walter Tower cardiothoracic suite.

**Table 3:** Effect of IBAI implementation on operating room outcomes. Difference-in-differences estimates comparing Walter Tower (intervention site) with a synthetic control composed of matched comparison sites before and after IBAI deployment. The coefficient on ‘Impact of IBAI on Walter Tower’ represents the estimated average treatment effect of IBAI. Overtime is defined as minutes past 3:00 p.m. for cases scheduled to end by that time. The second column reports log-transformed outcomes; all other outcomes are in levels.

	Number of Cases per Month	Number of Cases per Month (Proportional)	Number of Open Rooms per Day	Number of Cases per Room per Day	Overtime Minutes past 3 p.m. per Room per Day	Median Late Start Minutes per Case
Impact of IBAI on Walter Tower	24.684 ***	0.069 **	0.269	0.035	−0.754	−2.717 *
Standard error	(7.625)	(0.024)	(0.235)	(0.040)	(1.018)	(1.501)
95% CI (unadjusted)	[8.330, 41.037]	[0.017, 0.122]	[−0.235, 0.773]	[−0.050, 0.119]	[−2.938, 1.430]	[−5.936, 0.503]
*p*-value (unadjusted/Bonferroni)	0.006/0.036 ****	0.014/0.081	0.272/>0.99	0.397/>0.99	0.471/>0.99	0.092/0.551
Walter Tower indicator	0.038	0	−0.004	−0.720 ***	0.978	−2.083
Standard error	(6.028)	(0.019)	(0.186)	(0.031)	(0.805)	(1.187)
Observations	32	32	32	32	32	32

* Significant at 10% level, ** significant at 5% level, *** significant at 1% level, **** Bonferroni-adjusted *p*-value significant at 5% level.

## Data Availability

The aggregated monthly site-level data supporting the findings of this study are not publicly available, as they remain subject to Houston Methodist institutional data governance requirements, but are available from the corresponding author on reasonable request, subject to institutional approval and any applicable data use agreement. The analysis code (synthetic control weights and difference-in-differences regression scripts) is available from the corresponding author on reasonable request.

## Supplementary Materials

The following supporting information can be downloaded at: https://www.mdpi.com/article/10.3390/jimaging12070283/s1, Table S1: Synthetic control donor weights by model specification; Table S2: Case duration and service-line composition at Walter Tower, pre- versus post-deployment; Table S3: Sensitivity of the estimated effect on monthly case volume to the length of the pre-deployment window; Figure S1: Monthly trajectories of all study outcomes at Walter Tower compared with the synthetic control; Figure S2: In-space permutation placebo gap plots for all six study outcomes.

## Abbreviations

The following abbreviations are used in this manuscript:

**Abbreviation**	**Definition**
AI	Artificial intelligence
ASC	Ambulatory surgery center
ASU	Ambulatory surgery unit
CI	Confidence interval
DMAIC	Define, Measure, Analyze, Improve, Control
EHR	Electronic health record
HMH	Houston Methodist Hospital
IBAI	Image-based artificial intelligence
IRB	Institutional Review Board
LSS	Lean Six Sigma
OR	Operating room
RMSPE	Root mean squared prediction error
SD	Standard deviation
WT	Walter Tower
YOLO	You Only Look Once
