# Supplementary material for: Implementation of Image-Based Artificial Intelligence Is Associated with Increased Case Volume in a High-Acuity, 15-Room Cardiothoracic Operating Suite at a Tertiary Academic Hospital
Source: J Imaging. 2026 Jun 27;12(7):283. doi: 10.3390/jimaging12070283 (PMC13412611; doi:10.3390/jimaging12070283)
Supplement: Supplementary file 1 [file jimaging-12-00283-s001.zip › Figure S1. Monthly trajectories of all study outcomes at Walter Tower compared with the synthetic control.pdf]

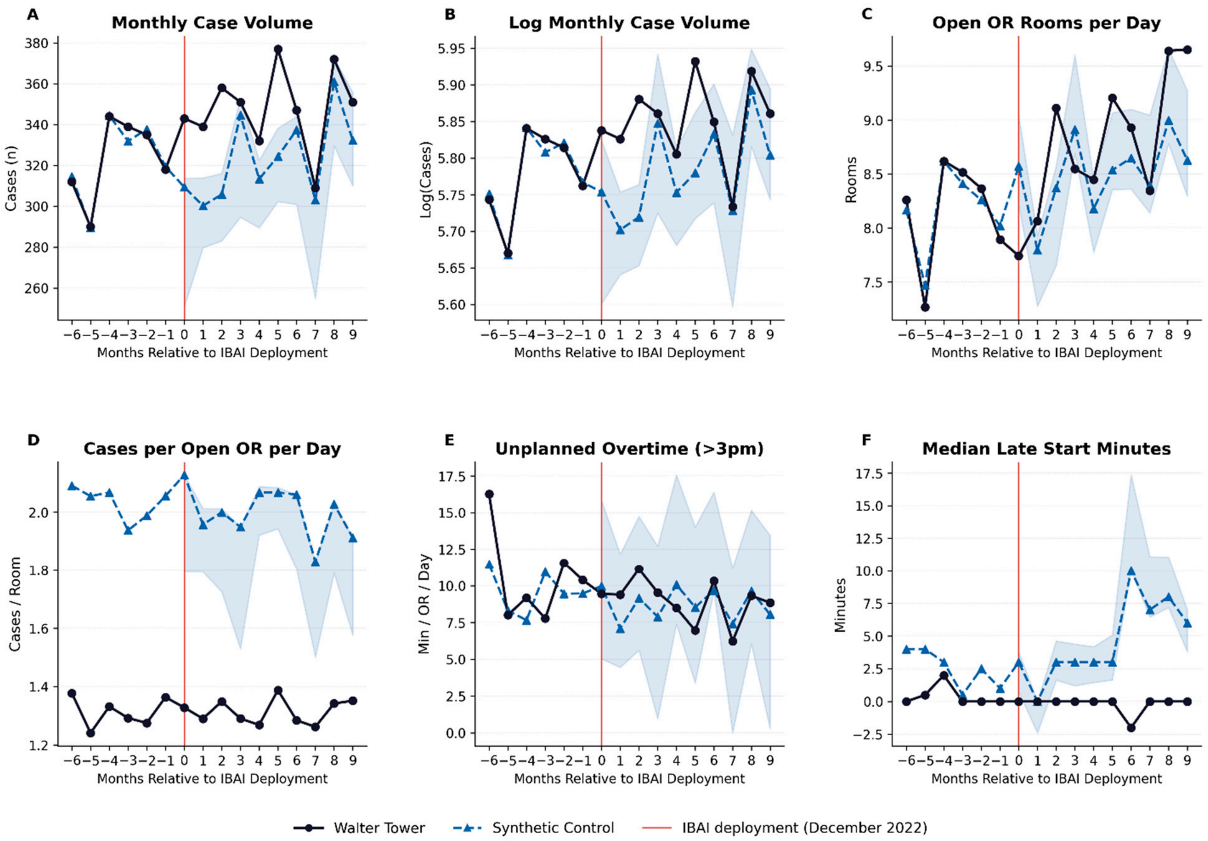

Supplementary Figure S1. Monthly trajectories of all study outcomes at Walter Tower compared with the synthetic control. Monthly trajectories are shown from 6 months before to 10 months after IBAI deployment. Panels: (A) monthly case volume; (B) log monthly case volume; (C) open operating rooms per day; (D) cases per open operating room per day; (E) unplanned overtime past 3:00 p.m. (minutes per open operating room per day); (F) median late start minutes per case. In each panel the solid line is Walter Tower, the dashed line is the synthetic control, and the vertical line marks the December 2022 IBAI deployment. The shaded band represents the plausible range of synthetic control predictions, based on the variability of the synthetic control's fit during the pre-deployment period. It is distinct from the shaded band in Figure 6 (monthly case volume), which instead shows the 5th to 95th percentile of the placebo distribution from the in-space permutation test.
