# Supplementary material for: Implementation of Image-Based Artificial Intelligence Is Associated with Increased Case Volume in a High-Acuity, 15-Room Cardiothoracic Operating Suite at a Tertiary Academic Hospital
Source: J Imaging. 2026 Jun 27;12(7):283. doi: 10.3390/jimaging12070283 (PMC13412611; doi:10.3390/jimaging12070283)
Supplement: Supplementary file 1 [file jimaging-12-00283-s001.zip › Figure S2. In-space permutation placebo gap plots for all six study outcomes.pdf]

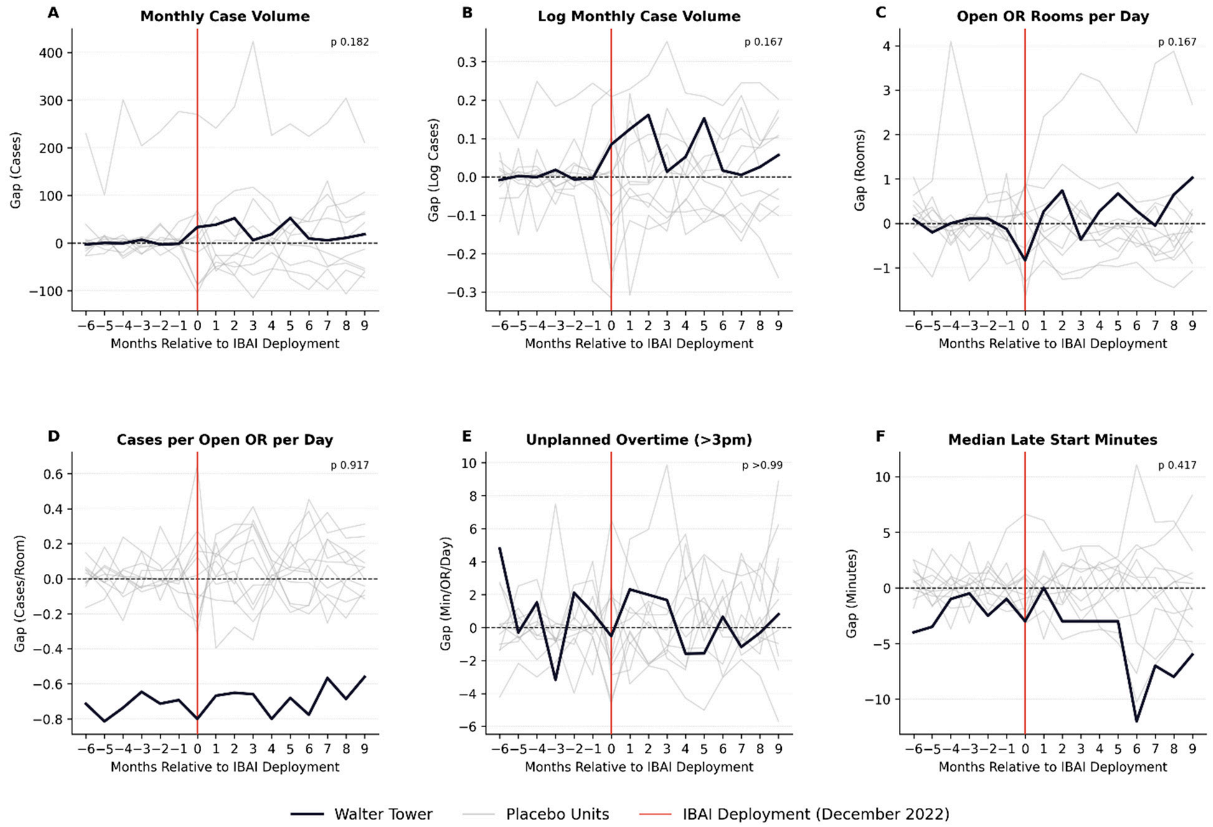

Supplementary Figure S2. In-space permutation placebo gap plots for all six study outcomes. Panels: (A) monthly case volume; (B) log monthly case volume; (C) open operating rooms per day; (D) cases per open operating room per day; (E) unplanned overtime past 3:00 p.m. (minutes per open operating room per day); (F) median late start minutes per case. In each panel, the line plots the gap between a site's observed outcome and its synthetic control, from 6 months before to 10 months after IBAI deployment. The solid dark line is Walter Tower; the gray lines are the placebo units, each obtained by treating one donor site in turn as the intervention site and estimating its synthetic control from the remaining donor sites. The vertical line marks the December 2022 IBAI deployment. The value shown in each panel is Walter Tower's empirical permutation p-value, defined by its rank on the ratio of post-deployment to pre-deployment root mean squared prediction error (RMSPE) relative to the placebo units. Panel A (monthly case volume) is based on 11 units (Walter Tower and 10 donor sites); fewer donor sites yielded converged synthetic controls in the raw case-volume specification than in the other outcomes, so this panel’s placebo distribution is smaller and its empirical p-value (2/11 = 0.182) has a denominator of 11 rather than 12. This does not affect the interpretation of the result.

In-space permutation (placebo) gap plots for all study outcomes (Panels A–F: monthly case volume, log case volume, open operating rooms per day, cases per open operating room per day, unplanned overtime past 3:00 p.m., and median late start minutes per case).
