# Supplementary material for: Implementation of Image-Based Artificial Intelligence Is Associated with Increased Case Volume in a High-Acuity, 15-Room Cardiothoracic Operating Suite at a Tertiary Academic Hospital
Source: J Imaging. 2026 Jun 27;12(7):283. doi: 10.3390/jimaging12070283 (PMC13412611; doi:10.3390/jimaging12070283)
Supplement: Supplementary file 1 [file jimaging-12-00283-s001.zip › Table S1. Synthetic control donor weights by model specification.pdf]

Table S1. Synthetic control donor weights by model specification. Weights for the full donor pool of 11 Houston Methodist comparison sites considered for the synthetic control group used to estimate Walter Tower's (WT) counterfactual case volume in the absence of IBAI deployment. Weights were estimated using the Python scpi_pkg package (version 2.2.7) to minimize the mean squared prediction error between WT and the weighted combination of donor sites during the 6-month pre-deployment period (June through November 2022). Weights are constrained to be non-negative and sum to one.

Donor Site	Weight (Case Volume, cases_n)	Weight (Log Case Volume, ln_cases_n)
HMH MAIN OR	0.36	0.34
HMH DUNN 6 OR	0.32	0.25
HMSJ OR	0.18	0.28
HMWB CF OR	0.10	0.11
HMH OPC 18 OR	0.04	9.04e-08
HMW OR	4.05e-09	0.03
HMWB OR	1.86e-09	1.28e-07
HMTW OR	6.10e-10	9.94e-08
HMSL MAIN OR	9.05e-11	2.60e-08
HMH DUNN OR	5.67e-11	1.79e-08
HMCL OR	3.87e-12	9.14e-09

Walter Tower (WT03) is the intervention site and is not part of the donor pool.

Abbreviations: HMH, Houston Methodist Hospital; HMCL, Houston Methodist Clear Lake; HMSJ, Houston Methodist San Jacinto; HMSL, Houston Methodist Sugar Land; HMTW, Houston Methodist The Woodlands; HMW, Houston Methodist West; HMWB, Houston Methodist Willowbrook; OPC, Outpatient Center; OR, operating room. Weights reflect the raw case count (cases_n) specification. The log-transformed model specification may select a different combination of donor sites.
