# Supplementary material for: Implementation of Image-Based Artificial Intelligence Is Associated with Increased Case Volume in a High-Acuity, 15-Room Cardiothoracic Operating Suite at a Tertiary Academic Hospital
Source: J Imaging. 2026 Jun 27;12(7):283. doi: 10.3390/jimaging12070283 (PMC13412611; doi:10.3390/jimaging12070283)
Supplement: Supplementary file 1 [file jimaging-12-00283-s001.zip › Table S2. Case duration and service-line composition at Walter Tower, pre- versus post-deployment.pdf]

Table S2. Case duration and service-line composition at Walter Tower, pre- versus post-deployment.

Measure	Pre-deployment (June through November 2022)	Post-deployment (December 2022 through September 2023)
Cases, n	1,938	3,479
Mean case duration, minutes (SD)	259.0 (144.6)	245.7 (141.8)
Median case duration, minutes [Q1, Q3]	221.0 [138.0, 374.8]	208.0 [128.0, 342.5]
Cardiothoracic, vascular, and thoracic service lines, % of cases	86	87

Case duration measured as wheels-in to wheels-out. Q1, Q3: first and third quartiles. The most frequent procedures were identical across periods and in nearly the same rank order.
