# Supplementary material for: Implementation of Image-Based Artificial Intelligence Is Associated with Increased Case Volume in a High-Acuity, 15-Room Cardiothoracic Operating Suite at a Tertiary Academic Hospital
Source: J Imaging. 2026 Jun 27;12(7):283. doi: 10.3390/jimaging12070283 (PMC13412611; doi:10.3390/jimaging12070283)
Supplement: Supplementary file 1 [file jimaging-12-00283-s001.zip › Table S3. Sensitivity of the estimated effect on monthly case volume to the length of the pre-deployment window.pdf]

Table S3. Sensitivity of the estimated effect on monthly case volume to the length of the pre-deployment window. In the log clean pre-period specification the synthetic control fit more parameters than pre-period observations, producing a near-perfect fit consistent with overfitting; this estimate should be interpreted cautiously.

**Outcome**	**Specification**	**Pre-deployment window**	**Pre-period months**	**Effect (coefficient)**	**p-value**	**Pre-period RMSPE**
Monthly case volume	Primary	June to November 2022	6	24.7	0.006	3.34
Monthly case volume	Clean pre-period	June to August 2022	3	15.1	0.245	3.34
Monthly case volume (log)	Primary	June to November 2022	6	0.069	0.014	0.009
Monthly case volume (log)	Clean pre-period	June to August 2022	3	0.102	0.007	<0.001

Clean pre-period excludes the September through November 2022 months that overlapped with camera installation and system training. RMSPE: root mean squared prediction error over the pre-deployment period.
